# Burnout prevalence and associated factors in medical students in integrated modular curriculum: A cross-sectional study

**DOI:** 10.12669/pjms.38.4.5052

**Published:** 2022

**Authors:** Khurram Irshad, Ifra Ashraf, Fahad Azam, Abida Shaheen

**Affiliations:** 1 Khurram Irshad, MBBS, FCPS. Associate Professor, Department of Physiology, Shifa College of Medicine, Shifa Tameer-e-Millat University, Islamabad, Pakistan; 2 Ifra Ashraf, MBBS, FCPS. Assistant Professor, Department of Physiology, Shifa College of Medicine, Shifa Tameer-e-Millat University, Islamabad, Pakistan; 3 Fahad Azam, MBBS, M. Phil., Ph.D. Associate Professor, Department of Pharmacology, Shifa College of Medicine, Shifa Tameer-e-Millat University, Islamabad, Pakistan; 4 Abida Shaheen, MBBS, M.Phil., Ph.D. Professor, Department of Pharmacology, Shifa College of Medicine, Shifa Tameer-e-Millat University, Islamabad, Pakistan

**Keywords:** Prevalence, Burnout, Stress, Medical education

## Abstract

**Objectives:**

The present study aimed to explore the prevalence of burnout syndrome and to find the association of burnout symptoms with sociodemographic factors in medical students.

**Methods:**

A cross-sectional study was conducted between September 2019 to January 2021 at Shifa College of Medicine, Islamabad. A total of 284 medical students from the first year to the fifth year participated in this cross-sectional study. After taking informed consent, students were asked to record responses on the Burnout Clinical Subtype Questionnaire BCSQ-12. In addition, a structured questionnaire was developed to obtain information about the sociodemographic status.

**Results:**

Prevalence of burnout was observed among day scholars, females, and students having a relatively sedentary lifestyle. Low family income showed a significant association of burnout in medical students (p=0.025). A significant association was found in the mean scores of neglect subtype of burnout with low family household income (*p* = 0.010). The mean scores of the overload subtype of burnout and daily duration of sleep also showed a significant association (*p* = 0.039).

**Conclusion:**

The prevalence of burnout was associated with low household income and sleep deprivation. Gender, academic year and physical activity did not have a significant association with burnout syndrome. The high prevalence of burnout syndrome necessitates appropriate interventions to identify and reduce the prevalence of burnout in medical students.

## INTRODUCTION

Undergraduate medical education is a highly challenging and demanding path that may predispose many students to an increased risk of developing burnout and stress. Burnout syndrome may significantly affect the learning capabilities of medical students which may cause physical and psychological disturbances.^1^

The term burnout is described as the loss of motivation or incentive in situations when desired results are not achieved despite devotion to a goal.^2^ Burnout induced chronic stress leads to physical and emotional tiredness, feeling of indifference and lack of achievements in individuals working in stressful conditions.^3^ The professional responsibilities of healthcare professionals make them more vulnerable to stress and anxiety due to little room for negligence in health care delivery. The mental health of medical students is of utmost importance to manage the burden of medical education in a high-stress environment.^4^,^5^

Burnout syndrome includes two important components, namely emotional exhaustion (EE) and depersonalization (DP); emotional exhaustion (EE) is explained as emotional insensitivity along with a low perception of individual achievement. Depersonalization is described as the absence of empathy, decreased motivation and increased sense of isolation.^6^

Recently, burnout has been described with the help of the “Burnout Clinical Subtype Questionnaire” (BCSQ-36) which distinguishes between the three clinical variants of burnout namely the frenetic, under-challenged and worn-out subtypes depending on the enthusiasm for work, lack of motivation, low appreciation and the overall working environment.^7^

Burnout syndrome does not only affect the learning abilities of medical students but also leads to physical, mental and social hazards like drowsiness, fatigue, eating disorders, migraine, emotional instability and drug abuse in extreme situations.^8^ The majority of the medical students in the UK and Australia have been found to be suffering from burnout during their medical education and revealed associated psychiatric disorders and suicidal ideation.^9^ Similarly, a high prevalence of burnout in students has also been reported in the public and private medical institutions of Pakistan.^10^,^11^

Despite the introduction of student-centred learning strategies, various factors such as communication with patients and interaction with other healthcare providers contribute to the prevalence of stress in medical students negatively affecting their physical and psychological health.^12^ The uncertainty about study behaviour, progress and aptitude have also been reported to be the main factors associated with stress in medical students.^13^

This scenario makes it important to identify the factors that cause burnout in medical students and take appropriate steps to minimize it. With this background, this study aimed to assess the prevalence of burnout syndrome and find the association of burnout symptoms with relevant sociodemographic factors among medical students in Pakistan.

## METHODS

A cross-sectional study was conducted between September 2019 to January 2021 at Shifa College of Medicine, Islamabad, Pakistan after taking approval from the Institutional Review Board (IRB # 002-492-2019). The teaching methodology comprises an active student-centred approach in an integrated modular curriculum including small group discussions (SGD), self-directed learning (SDL), problem-based learning (PBL), large group interactive sessions (LGIS) and clerkships.

A total of 284 students from the first year to the fifth year participated in the study. Students who did not provide informed consent to be a part of the study were not included. The study participants provided responses in a structured questionnaire for sociodemographic status. The sociodemographic variables included gender, marital status, academic year, residence financial status, smoking, sleeping hours and physical activity. All the participants were also asked to record responses in the Burnout Clinical Subtype Questionnaire”, BCSQ-12 adapted for use with students (“Burnout Clinical Subtype Questionnaire Students Survey”, or BCSQ-12-SS).^14^

The three clinical variants of burnout are the frenetic, under-challenged and worn-out subtypes where the “frenetic” subtype of burnout is described as stress in ambitious and overworked individuals who invest a significant proportion of time for their profession. The “under-challenged” subtype of burnout is described as stress in individuals who often perform mechanical tasks and is characterized by the feeling of lack of progression in addition to the feeling of indifference and boredom. The “worn-out” subtype of burnout is characterized by a feeling of the lack of appreciation and perception of less control over outcomes and results in a lack of interest in work responsibilities.^7^

### Statistical Analysis:

The SPSS latest Statistics version was used for data analysis. Student’s t-test and ANOVA were applied for comparison between groups for quantitative variables; continuous variables were presented in mean±SD; categorical variables were presented by frequencies and percentages and were analyzed by the Chi-square and Fisher’s Exact test. A p-value <0.05 was considered significant.

## RESULTS

A total of 284 students recorded their responses. The frequencies of different sociodemographic factors of the study participants are summarized in Table-I. The majority of the study participants belonged to the first three years. Out of 284 students, 69% took adequate sleep of more than six hours and 67% had a relatively sedentary lifestyle and exercised less than three times a week.

**Table-I:** Sociodemographic data of medical students.

Variables	n (%)
***Gender***	
Male	122(43%)
Female	162(57%)
***Marital status***	
Single	277(97.5%)
Married	7(2.5%)
***Year of study***	
First-year	74(26.1%)
Second-year	93(32.7%)
Third-year	62(21.8%)
Fourth-year	17(6%)
Final-year	38(13.4%)
***Residence***	
Day scholar	201(71%)
Hostilities	81(29%)
***Financial status***	
Low income (< Rs: 50,000 per month)	9(3.4%)
Middle income (Rs: 50,000 -100,000 per month)	43(16%)
High income (> Rs: 100,000 per month)	216(80.6%)
***Smoking***	
Smokers	13(4.6%)
Non smokers	265(95.3) %
***Sleeping hours***	
More than 6 hours	193(69.4%)
Less than 6 hours	85(30.6%)
***Work out***	
More than 3 times/week	92(32.7%)
Less than 3 times/week	189(67.2%)

The association of different demographic variables with high and low scores of burnout among medical students are shown in Table-II. Participants with >75th percentile for any dimension of the questionnaire were assigned high scores, while those scoring <75th percentile were categorized as low scorers.^14^ Chi-square analysis revealed that students with a low household income have a higher prevalence of burnout as compared to high-income groups with a high statistical significance (*p*=0.020). The prevalence of burnout among females, day scholars, students with a relatively sedentary lifestyle and sleep deprivation was not statistically significant.

**Table-II:** Association of different demographic variables with high and low scores of burnouts.

Variables	High Scores >75 percentile	Low scores <75 percentile	p-value
***Gender***			
Male	33(11.61%)	89(31.33%)	.418
Female	51(17.95%)	111(39.08%)	
***Marital status***			
Single	82(28.87%)	195(68.66%)	.953
Married	2(0.70%)	5(1.76%)	
***Year of study***			
First-year	14(4.93%)	60(21.13%)	.202
Second-year	33(11.62%)	60(21.13%)	
Third-year	19(6.69%)	43(15.14%)	
Fourth-year	6(2.11%)	11(3.87%)	
Final-year	12(2.23%)	26(9.15%)	
***Residence***			
Day-scholar	64(22.70%)	137(48.58%)	.191
Boarders	19(6.74%)	62(21.99%)	
***Financial status***			
Low-income	4(1.49%)	5(1.87%)	.020*
Middle-income	20(7.46%)	23(8.58%)	
High-income	57(21.27%)	159(59.33%)	
***Work out per week***			
More than 3 times	24(8.54%)	68(24.20%)	.329
Less than 3 times	60(21.35%)	129(45.91%)	
***Smoking status***			
Smoker	4(1.44%)	9(3.24%)	.276
Nonsmoker	80(28.78%)	185(66.55%)	
***Sleeping hours***			
More than 6 hours	55(19.78%)	138(49.64%)	.456
Less than 6 hours	28(10.07%)	57(20.50%)	

The analysis of the socio-demographic factors and mean values of total burnout scores along with mean scores of different subtypes of burnout namely lack of development, overload, and neglect are shown in Table-III. Results show a significant association between monthly income and mean scores of burnout (*p*=0.025); a significant association was also seen with a mean score of neglect (*p*=0.010). The mean scores of overload and daily duration of sleep showed a significant association (*p*=0.039).

**Table-III:** Analysis for mean values of total burnout score: neglect, overload and lack of development.

Variables (n)	Total score		Overload		Lack of development		Neglect	

	Mean±SD	p-value	Mean±SD	p-value	Mean±SD	p-value	Mean±SD	p-value
***Gender***								
Male(122)	40.96±9.50	.519	14.22±4.8	.484	14.73±4.09	.886	12.01±4.53	.178
Female(162)	41.67±8.87		14.33±4.8		14.65±4.56		12.68±3.82	
***Marital status***								
Single(277)	41.37±9.16	.916	14.32±4.8	.386	14.64±4.34	.248	12.41±4.13	.663
Married(7)	41±8.66		21.72±6.4		16.57±4.93		11.71±4.89	
***Level of course***								
Year 1(74)	40.22±8.06	.435	14.54±4.3	.572	14.05±3.78	.253	11.62±4.0	.283
Year 2(93)	41.91±10.6		13.97±5.3		14.96±4.68		12.99±4.4	
Year 3(62)	42.21±7.27		14.74±4.6		14.85±3.32		12.61±4.1	
Year 4(17)	43.41±8.72		15.18±4.2		16.47±5.85		11.76±2.5	
Year 5(38)	39.95±9.99		13.42±5.2		14.18±5.20		12.34±4.1	
***Residence***								
Day-Scholar (201)	41.66±9.01	.335	14.43±5.03	.324	14.79±4.57	.434	12.43±4.03	.881
Hostelite (81)	40.49±9.50		13.80±4.36		14.34±3.83		12.34±4.48	
***Financial status***								
Low income (9)	45.66±11.88	.025*	15.78±5.58	.288	15.89±14.8	.533	14±4.69	.010*
Middle income (43)	44.16±8.04		15.12±4.77		15.16±4.20		13.88±3.89	
High income (216)	9.06±.62		14.07±4.88		14.58±4.46		12.02±4.02	
***Workout***								
>3/week(92)	40.80±8.63	.457	13.77±4.83	.221	14.67±4.27	.935	12.36±4.18	.895
<3/week(189)	41.67±9.42		14.52±4.82		14.72±4.43		12.43±4.16	
***Smoking***								
Yes(13)	38±12.65	.175	13.23±6.26	.434	13.15±5.73	.182	11.61±3.66	.499
No(265)	41.55±9.01		14.32±4.80		14.82±4.31		12.42±4.20	
***Daily sleep hours***								
≥six(193)	41.08±9.12	.49	13.91±4.84	.039*	14.71±4.36	.846	12.46±3.89	.557
<six(85)	41.95±9.43		15.21±4.73		14.60±4.25		12.14±4.75	

## DISCUSSION

The acknowledgement of burnout as a public health problem directed scientists to find out the factors that lead to work-related diseases and to find out ways to fulfil physical, sensitive and community-based needs arising from the onset of burnout. This scenario suggests the need for scientific studies to explore the prevalence of burnout and relevant factors in different populations with a special focus on medical undergraduate students.^15^-^17^

According to our findings, a higher prevalence of burnout was observed in females, day scholars, students with sleep deprivation and sedentary lifestyle but the association was not statistically significant. These findings are in agreement with a study conducted by Shah et al, according to which the prevalence of stress was significantly higher in females and in students who reported difficulty in sleeping; however, this study did not show a significant association of stress with low household income which is in contrast to our findings.^11^ Another study by Altannir et al conducted in Saudi Arabia reported that the prevalence of overall burnout was more common in female medical students; this study further reported that female students suffered from a low sense of personal progression, emotional exhaustion and depersonalization in comparison to male students and the prevalence of burnout reduced as the students progressed to higher classes.^1^ Our findings are not in agreement with these findings as our study showed a similar level of stress in all academic years. Other studies reported a higher prevalence of stress in the early years and a similar prevalence of stress in both genders. These findings are contradictory to our findings in which stress was similar in all academic years and females suffered more stress as compared to male counterparts.^18^,^19^

According to the results of our study, the prevalence of stress is significantly higher among students belonging to families with low household income; these results are in agreement with the findings reported by other researchers showing a significant association of the prevalence of stress among students belonging to families with low household income. Possible reasons for these findings could be stress induced by the inability to access books, handouts and other basic requirements. Furthermore, differences in culture, region, curriculum, diversity, teaching experience of faculty members, the level of care provided to the students and the differences in the instruments could be the reasons for stress among medical students in the reported studies.^20^,^21^

Our study showed that sleep deprivation is one of the important factors which leads to burnout in medical students which is also analyzed by different studies reporting that reduction in both sleep quality and quantity is linked with poor health outcomes leading to depression, anxiety, and feeling of self-pity. In a recently conducted study in Pakistan, emotional exhaustion was found to be the main contributor to burnout among medical students.^22^ Poor sleep quality may further reduce students’ ability to cope with emotional challenges thus good practices and behaviours promoting good quality sleep might be beneficial to minimize stress and burnout.^22^-^24^

### Limitations of the study:

The main limitation of our study is that we could not investigate the interventions used by the medical students as coping strategies to minimize their burnout. Another limitation is the lack of focused group sessions to further explore possible reasons for burnout.

## CONCLUSION

The prevalence of burnout is high in medical students with low household income and sleep deprivation. Further research is required to investigate the influence of burnout on academic performance and innovative strategies should be planned to reduce burnout in medical students.

### Authors Contribution:

**KI:** Conceived and designed the study, collected data, drafted and reviewed the final manuscript draft.

**IA:** Designed the study, collected data, drafted and reviewed the final manuscript draft.

**FA & AS:** Analyzed data, drafted manuscript and reviewed final manuscript draft.
